# Protocol for the generation and purification of minicells from *Lactiplantibacillus plantarum*

**DOI:** 10.71150/jm.2412002

**Published:** 2025-05-27

**Authors:** Hyemin Kang, Donghyun Kim, Juhyun Kim

**Affiliations:** 1 School of Life Sciences, BK21 FOUR KNU Creative BioResearch Group, Kyungpook National University, Daegu 41566, Republic of Korea; 2 Department of Microbiology and Immunology, Seoul National University College of Medicine, Seoul 03080, Republic of Korea; 3 Department of Biomedical Sciences, Seoul National University College of Medicine, Seoul 03080, Republic of Korea; 4 Institute of Endemic Diseases, Seoul National University Medical Research Center, Seoul 03080, Republic of Korea

**Keywords:** *L. plantarum*, minicells, MinD

## Abstract

Minicells, which are anucleate cells generated by irregular cell division, are emerging as promising drug delivery systems owing to advances in synthetic biology. However, their development is largely limited to a few model bacteria, highlighting the need to explore minicell platforms in alternative hosts. *Lactiplantibacillus plantarum* (*L. plantarum*), a probiotic bacterium classified as Generally Recognized as Safe, is an ideal candidate for such exploration. Minicell-producing *L. plantarum* was engineered by deleting the putative *minD* gene via plasmid-mediated homologous recombination, which inactivates cell division to form spherical minicells. Anucleate cells were isolated through differential centrifugation and filtration, followed by additional drug treatment to completely eliminate progenitor cells. Microscopy and flow cytometry analyses of the purified sample confirmed the absence of progenitor cells by DAPI staining. This protocol effectively produces bacterial minicells from *L. plantarum* for use in various biotechnological applications, including therapeutic agent delivery.

## Overview

Bacterial genetic engineering for clinical purposes has advanced into a refined platform for targeted therapeutic delivery (Duong et al., 2019; Faghihkhorasani et al., 2023; Hosseinidoust et al., 2016). However, challenges such as unpredictable immune responses, potential off-target effects, and inconsistent efficacy and safety across diverse patient populations (Elowitz et al., 2002; Fooladi et al., 2023; Hodgman and Jewett, 2012; Kwok, 2010) limit its broad application. Advancements in design and control are needed to minimize risks and optimize therapeutic outcomes (Charbonneau et al., 2020). To overcome these challenges, bacterial minicells have been developed as an alternative chassis for drug delivery systems. Nano-sized minicells (100–400 nm diameter) are occasionally formed naturally from aberrant cell division, retaining most cellular components of the parent cell but lacking chromosomal DNA (Farley et al., 2016; Ni et al., 2021). While retaining functional capabilities, including intact cellular structures and intracellular components, minicells cannot reproduce, ensuring their safe therapeutic applications (Adler et al., 1967; de Boer et al., 1989). Minicells formation can also be induced by inactivating cell division (Ali et al., 2020; de Boer et al., 1989), which relies on MinCDE protein oscillation directing FtsZ to form the contractile Z-ring at the midcell, the optimal site for division. Disrupting the Min system facilitates minicell generation in various bacteria, including *Salmonella enterica* (Carleton et al., 2013), *Pseudomonas aeruginosa* (MacDiarmid et al., 2007), and *Bacillus subtilis* (Feddersen et al., 2021).

The homologous *minD* gene, found in various bacteria (Rothfield et al., 2005), can be deleted to produce minicell-generating strains with uncharacterized division systems (Carleton et al., 2013; de Boer et al., 1991; Lee et al., 2015; Marston and Errington, 1999). In this study, the *minD* gene of *Lactobacillus plantarum* WJL was deleted by using a constructed suicide plasmid, pGID023-LR, containing homologous regions flanking *minD*. Using a homologous recombination approach, a *minD*-deleted strain was engineered by introducing the plasmid into cells. Microscopy analysis revealed that the Δ*minD* strain produced elongated cells and small spherical minicells. The minicells were purified through differential centrifugation based on size and density, followed by ceftriaxone treatment to remove parent cells. DAPI staining indicated the absence of chromosomal DNA in the isolated minicells, which demonstrated therapeutic potential. Furthermore, the protocol outlined in this study can be applied to other non-model bacterial species, leveraging their distinct characteristics for specialized applications.

## Applications

Engineered minicells are increasingly used in drug delivery systems because of their high biocompatibility, minimized drug leakage, reduced toxicity, and enhanced drug-loading efficiency (Ali et al., 2020), which have inspired their development for diverse applications. For example, engineered *E. coli* minicells targeting cancer cells successfully delivered doxorubicin, resulting in significant tumor regression with lower doses in mouse models (MacDiarmid et al., 2007). Similarly, *E. coli* minicells with type IV secretion systems can transfer DNA and the anucleate minicells were further engineered to suppress specific bacterial species (Li et al., 2023). Furthermore, *S. typhimurium* minicells equipped with type III secretion systems delivered antigens, inducing MHC class I-restricted immune responses and activating CD8+ T-cells in vitro (Carleton et al., 2013).

The production of minicells by disrupting the bacterial cellular division system, as outlined above, has been studied but remains limited to a few model bacterial species. To expand to alternative hosts, we developed a strategy targeting the inactivation of a conserved bacterial gene responsible for cell division. This method enables the creation of minicells tailored to specific applications by using hosts with desirable traits.

**Fig. 1.**
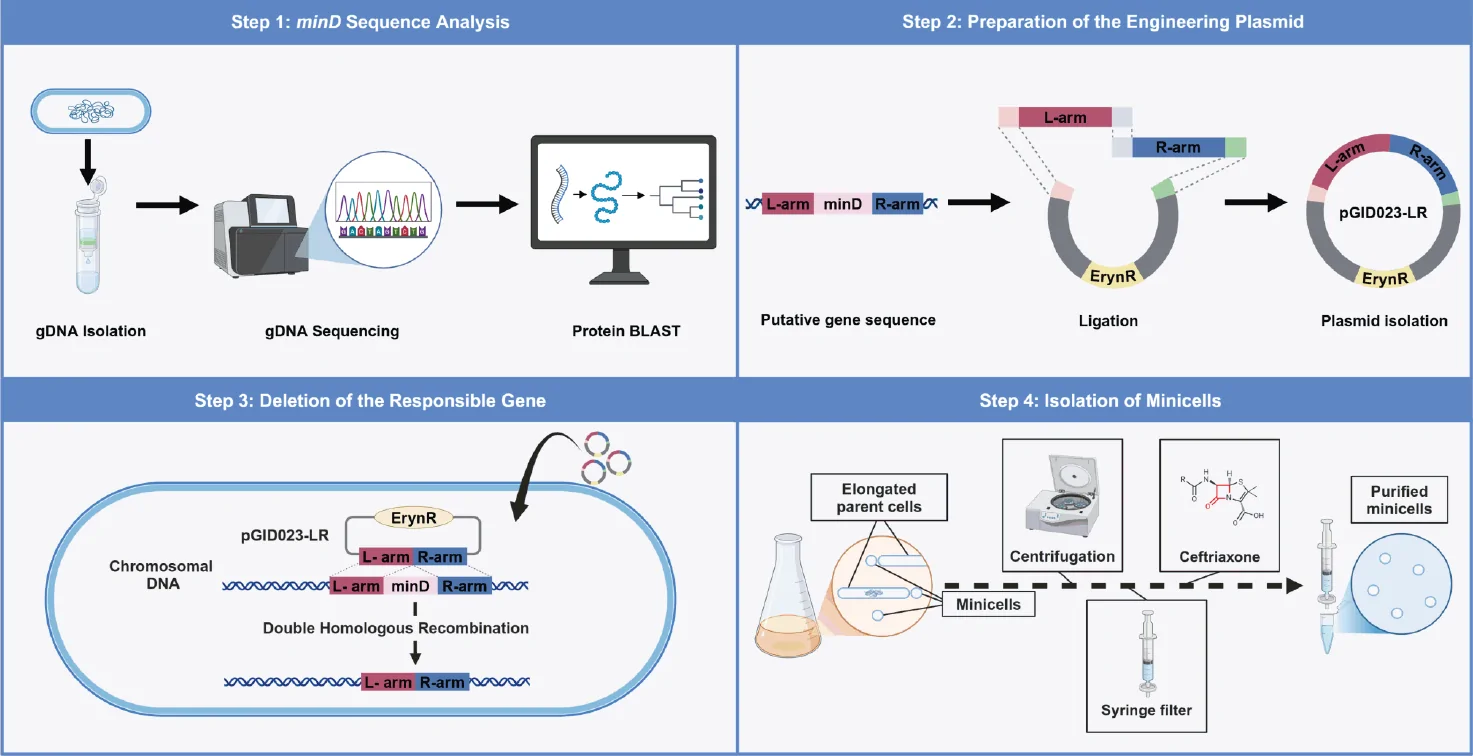
Overview of minicell production from *L. plantarum*. This figure summarizes the process of producing and purifying *Lactiplantibacillus plantarum* WJL-derived minicells in four steps. (1) Genomic DNA (gDNA) is extracted, sequenced, and analyzed to identify the *minD* gene. (2) A delivery plasmid is constructed with flanking homology regions of *minD*. (3) The *minD*-deficient strain was generated via a seamless allelic replacement approach. (4) Minicells were purified from the *minD*-deficient strain through differential centrifugation, filtration, and ceftriaxone treatment to remove parent cells, yielding minicells incapable of division.

This protocol describes the deletion of the putative *minD* gene in *Lactobacillus plantarum *(Fig. 1). Although previously uncharacterized, the gene was identified through genome sequencing and computational strain analysis. To inactivate the gene, a constructed plasmid carrying homologous arms flanking the target gene was introduced into the strain to induce homologous recombination. This approach enabled precise gene removal without leaving any scars or markers in the chromosome. Minicells emerged from the *minD*-deleted strain, indicating that the gene is involved in cellular division. Additionally, this protocol is an optimized method for purifying *L. plantarum*-derived minicells using differential centrifugation and antibiotic treatment to effectively eliminate nucleated parent cells.

Minicells derived from this strain offer distinct advantages, leveraging the inherent beneficial properties of probiotics. As Generally Recognized as Safe organisms, *Lactobacillus* strains provide a safe and biocompatible platform (Huang et al., 2022; Masood et al., 2011), reducing the risk of adverse immune reactions often associated with pathogenic bacteria. *L. plantarum* stands out among *Lactobacillus* species due to its genetic flexibility, environmental robustness, and probiotic safety, making it a preferred chassis for engineering in a variety of applications (de Vries et al., 2006). Moreover, the strain can strongly adhere to intestinal epithelial cells and interact with the host immune system (Garcia-Gonzalez et al., 2018), providing unique opportunities for engineering *L. plantarum* as a vehicle for immunomodulatory applications, such as vaccine delivery or therapeutic modulation of gut inflammation. Owing to these properties, *L. plantarum* minicells exhibit immune-stimulatory properties, activating innate immunity and enhancing vaccine efficacy through their adjuvant effects (Kawashima et al., 2011; Kuczkowska et al., 2019). Their natural association with mucosal surfaces, such as the gut and respiratory tract, makes them ideal for targeted delivery of drugs, vaccines, or bioactive molecules (Adlerberth et al., 1996; Wang et al., 2016). Additionally, these minicells can be engineered to withstand harsh conditions such as low pH or bile salts, making them ideal for oral delivery systems (de Vries et al., 2006; Razavi et al., 2021). Their established use in fermentation industries ensures cost-effective and scalable production, while their genetic tractability allows functional customization for specific therapeutic or industrial needs (Son and Jeong, 2020; Wu et al., 2021). These features make *Lactobacillus* minicells a safe, versatile, and efficient delivery platform for various biomedical applications (Ijaz et al., 2024).

## Methods

### Culture conditions

Unless otherwise indicated, bacteria cells were routinely grown at 37°C in Luria-Bertani or Man-Rogosa-Sharpe (MRS) medium for *E. coli* and *L. plantarum*, respectively (Table 1). Bacteria were cultured in 15-ml test tubes with shaking at 170 rpm with 2 ml of the medium specified for the corresponding experiment. Whenever necessary, erythromycin or ceftriaxone was added to cell cultures to ensure plasmid retention or eliminate nucleated parent cells.

### Identification of the homolog for cellular division in *L. plantarum*

The MinD protein, a critical regulator of bacterial cell division, plays a key role in preventing FtsZ ring formation at the cell poles (Rothfield et al., 2005; Yamaichi and Niki, 2000). Deleting the *minD* gene disrupts this regulation, causing asymmetric cell division characterized by septum formation at the cell poles rather than at the midcell. This facilitates minicell production (Carleton et al., 2013; de Boer et al., 1991). To generate minicells from *Lactobacillus plantarum*, the *minD* gene was targeted. Although the draft genome sequence of *L. plantarum* strain WJL has been previously reported (Martino et al., 2015), a comprehensive sequence analysis was performed to ensure accuracy and identify potential genomic elements. Genomic DNA from *L. plantarum* WJL was extracted, and Nanopore sequencing was used to obtain sequence information. Through this analysis, a *minD* gene homolog was identified, corroborated by protein BLAST approaches, with its amino acids showing over 60% similarity to homologs in other bacteria, including *B. subtilis*, *E. coli*, and *Pseudomonas* spp. (Table 2).

### Genetic modification of *L. plantarum*

**Fig. 2.**
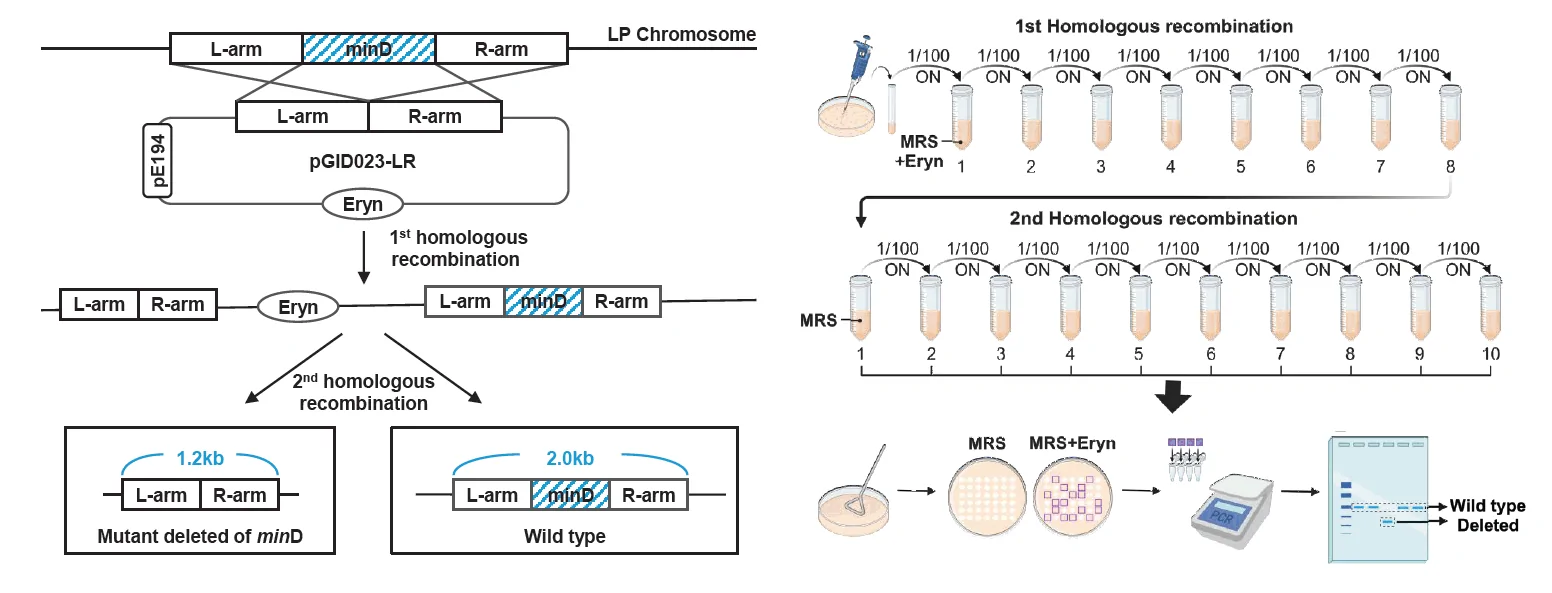
Simplified schematic of the genetic modification process in *L. plantarum*. The pGID023 plasmid, containing the upstream and downstream flanking regions of the *minD* gene, was constructed and introduced into *Lactiplantibacillus plantarum* WJL. The first recombination, randomly occurring at one flanking region, integrates the plasmid into the chromosomal DNA after several passages in an MRS medium supplemented with 5 μg/ml erythromycin. The second recombination, randomly occurring at one flanking region and involving intrachromosomal recombination at either the L-arm or R-arm region, was achieved by chance after 10 passages in an MRS medium without erythromycin. The final recombination yielded two potential outcomes: either a *minD*-deleted mutant strain or reversion to the wild-type strain.

To delete the target gene in *Lactobacillus plantarum* WJL, we employed a seamless allelic replacement approach (Hols et al., 1994). The delivery plasmid (pGID023-LR) used for deleting the *minD* gene was constructed using the shuttle vector, pGID023 (Hols et al., 1994), compatible with both *E. coli* and *L. plantarum*. This vector, derived from pJDC9, incorporates pE194 replication functions (Gryczan et al., 1982) and serves as an unstable integration vector conferring erythromycin resistance (Hols et al., 1994). To maintain the plasmid within *E. coli*, cultures were supplemented with erythromycin (200 μg/ml). The upstream (L-arm; 0.6 kb) and downstream (R-arm; 0.6 kb) regions of *minD* were amplified with primer pairs L-arm-fw/rv and R-arm-fw/rv, respectively (Table 3). These amplicons were joined via splice overlap extension PCR (SOEing PCR) (Horton et al., 2013) using primer pairs L-arm-fw and R-arm-rv (Table 3). The resulting PCR product was cloned as HinDIII-BamHI fragments in the pGID023 vector, forming plasmid pGID023-LR, which was kept in *E. coli* strain DH5α. This plasmid was electroporated into the genome of *L. plantarum*, and cointegration was achieved after eight passages in MRS medium supplemented with 5 μg/ml erythromycin. Campbell-type integration inserted the plasmid into the chromosome via the L-arm and R-arm. After 10 passages in MRS medium without erythromycin, intrachromosomal recombination excision at either the L-arm or R-arm region was achieved, yielding either the wild-type or *minD*-deficient phenotype with equal probability. The genotype was determined using the primer pair L-arm-fw and R-arm-rv (Fig. 2). The validated *minD* deletion mutant was designated as *Lactobacillus plantarum* Δ*minD* (Table 1).

### Sample preparation for microscopy

To characterize minicell-producing cells, the Δ*minD* strain was cultured overnight in MRS medium, diluted 100-fold, and regrown to the stationary phase. Culture samples (1 ml) were washed twice with phosphate-buffered saline (PBS) and finally resuspended in 200 µl PBS. The prepared cell suspension (2 µl) was subsequently placed on 0.01% poly-L-lysine (Sigma-Aldrich)-coated coverslips and dried. Chromosomes were stained by adding 250 µl of 4',6-diamidino-2-phenylindole (DAPI; 1 µg/ml) to the immobilized cells on the slip and incubated for 15 min at room temperature. After removing the DAPI solution, the coverslip was assembled on a slide with ProLong^TM^ Gold Antifade Mountant to prevent photobleaching and sealed using clear nail polish. Microscopy was performed using an Olympus BX53 apparatus equipped with a 100x phase-contrast objective and a fx900c camera. DAPI signals were detected using field-wide excitation with PE300. The images obtained were analyzed using the ImageJ software. Flow cytometry analysis was also performed to quantify DAPI-stained signals at the single-cell level.

## Materials

### A. Biological materials

All experiments described were conducted using *L. plantarum* WJL strain (Kim et al., 2013). *E. coli* DH5α was used as the transformation host for plasmid construction (Grant et al., 1990). Table 1 provides detailed information regarding the strain and plasmid characteristics.

### B. Reagents

● Tris-HCl Solution, pH 8.0 (T&I, BTH-9180-500mL)

● EDTA buffer (Aladdin, AL-E196386.0001)

● Triton® X-100 (HANLAB, HC0694-500ML)

● Lysozyme (Sigma-Aldrich, 10837059001)

● Phosphate-buffered saline (Dyne Bio, CBP3070)

● Sucrose (DUKSAN, 848)

● MgCl_2_ (DUKSAN, 2142)

● Glycine (Sigma-Aldrich, G8898)

● dH₂O (pH ≥ 7.0)

● Ethanol (SAMCHUN PURE CHEMICAL, E0235)

● Erythromycin (Sigma-Aldrich, E5389)

● MRS broth (BD Difco, 288130)

● MRS agar (BD Difco, 288210)

● HindIII restriction enzyme (NEB, R3104S)

● BamHI restriction enzyme (NEB, R3136S)

● T4 ligase (NEB, M0202S)

● ProLong^TM^ Gold Antifade Mountant (Thermo Fisher, P36984)

● 4',6-diamidino-2-phenylindole (TCI, D5888-1SET)

● Glycerol (DUKSAN, 56-81-5)

● Ceftriaxone Sodium (Merck, PHR1382)

● DNeasy Blood & Tissue Kit (Qiagen, 69504)

● MiniPrep Kit (GeneAll, 101-102)

### C. Consumable

● 1.5 ml microcentrifuge tubes (Axygen, MCT-150-C)

● 15 ml conical tubes (SPL, 50015)

● 50 ml conical tubes (SPL, 50050)

● 2 mm electroporation cuvettes (Thermo Fisher, 5520PK)

● 10 ml syringe (Bukwang Pharmaceutical, DM4201791700)

● 0.8 µm syringe filter (ADVANTEC, AD.25CS080AS)

### D. Equipment

● Microcentrifuge

● Refrigerated centrifuge

● Shaking incubator

● Electroporator

● NanoDrop spectrophotometer

● Thermal cycler

● Gel electrophoresis setup

● Vortex mixer

● Heating block

● -20℃ refrigerators

● -80℃ freezers

### E. Probe Primers

● L-arm-fw primer

● R-arm-rv primer

## Protocols

### A. *minD* sequence analysis

#### A-1. Genomic DNA extraction

Genomic DNA from *L. plantarum* was extracted using the Qiagen DNeasy Blood & Tissue Kit, with slight adjustments to enhance efficiency.

● Note: The kit offers optimized protocols that enable high-yield DNA extraction from various sample types, including blood, tissue, and both Gram-positive and Gram-negative bacteria.

1. A single colony of *L. plantarum* WJL strain was inoculated into 2 ml of MRS broth and incubated overnight at 37°C.

2. Cells were harvested by centrifuging the culture in a microcentrifuge tube at 7,197 × g for 10 min. Discard the supernatant.

3. The bacterial pellet was resuspended in 180 µl enzymatic lysis buffer (20 mM Tris·Cl, pH 8.0, 2 mM sodium EDTA, 1.2% Triton^®^ X-100, lysozyme) and incubated at 37°C for 30 min.

● Note: Lysozyme was added immediately before use to a final concentration of 30 mg/ml.

● Note: The heating block was preheated to prepare for the incubation in step 5.

4. Proteinase K (20 µl) and Buffer BL (200 µl) were added and thoroughly mixed by vortexing.

5. The mixture was incubated at 56°C for 30 min.

6. The tube was briefly spun down to remove any drops from inside the lid.

7. Ethanol (200 µl) was added to the sample, and a pulse vortex was used to mix the sample thoroughly.

● Note: The solution was thoroughly mixed to achieve homogeneity. If a white precipitate is formed, the entire mixture, including the precipitate, is carefully transferred to the DNeasy Mini spin column to prevent any loss.

8. The mixture from step 7, including any precipitate, was transferred into the DNeasy Mini spin column positioned in a provided 2 ml collection tube, centrifuged at 11,400 × g for 1 min, and the flow-through and collection tubes were discarded.

9. The collection tube was replaced with a new one, and 500 µl Buffer AW1 was added and centrifuged at 11,400 × g for 1 min. The flow-through and collection tubes were discarded.

10. Buffer AW2 (500 µl) was added and centrifuged for 3 min at 11,400 × g. The flow-through and collection tubes were discarded.

● Note: After centrifugation, the collection tube was carefully removed to avoid contact with the flow-through, which could result in ethanol carryover. If ethanol carryover occurs, the flow-through is discarded, the collection tube is reused, and centrifugation is repeated for 1 min at 11,400 × g.

11. The washing step from Step 10 was repeated.

12. The membrane was dried by incubating at room temperature for at least 15 min.

● Note: The membrane was thoroughly dried to prevent residual ethanol from interfering with subsequent reactions.

13. The collection tube was replaced with a clean 1.5 ml microcentrifuge tube and dH_2_O (100 µl) pipet directly onto the membrane.

● Note: Elution with 100 µl increases the final DNA concentration in the eluate but also decreases the overall DNA yield.

● Note: To elute DNA using dH_2_O, the pH of the water should be at least 7.0, as deionized water from some sources may be acidic.

● Note: For long-term storage of DNA, elution in Buffer AE is recommended since DNA stored in water is subject to acid hydrolysis.

14. Incubate at room temperature for 1 min, and then centrifuge for 1 min at 11,400 × g to elute.

● Note: Ensure that the dH_2_O is dispensed directly onto the center of the membrane for optimal elution of DNA.

● Note: Ensure the incubation step is completed before centrifugation to maximize DNA recovery during elution.

15. The concentration and purity of the isolated DNA was evaluated using a spectrophotometer.

#### A-2. DNA sequencing and analysis

1. The extracted genomic DNA was sent to a sequencing service provider (Plasmidsaurus) for whole-genome sequencing.

2. Upon receiving the sequencing data with bioinformatic analysis for genome annotation, the putative MinD protein-encoding encoding genes (membrane-associated ATPase) were identified, and the sequence was obtained.

3. Using the identified target sequence, a Protein BLAST analysis (NCBI BLAST) was performed to compare the query sequence from *L. plantarum* with sequences from other bacterial species. The resulting similarity scores were analyzed to confirm sequence homology.

### B. Preparation of the engineering plasmid

1. Upstream (L-arm; 0.6 kb) and downstream (R-arm; 0.6 kb) regions of the *minD* gene were amplified using L-arm-fw/rv and R-arm-fw/rv, respectively (Table 3).

● Note: The primers L-arm-rv or R-arm-fw contain a 20 bp overlapping region. The overlapping region should be maintained within a range of 20–30 bp. Additionally, a restriction enzyme site was included at the 5′ ends to design primers for L-arm-fw and R-arm-rv.

2. The amplified L-arm and R-arm fragments were recombined through their overlapping regions using L-arm-fw and R-arm-rv with SOEing PCR.

3. The recombined LR fragments and pGID023 plasmid were digested with HindIII and BamHI restriction enzymes, before being ligated using T4 ligase.

● Note: Alternatively, other DNA assembly techniques, such as Isothermal Assembly or Uracil Assembly, can be used to construct the recombinant plasmid. These methods require the design of primers specific to the chosen approach. Notably, they bypass the need for PCR, DNA digestion, and ligation steps, thereby streamlining the plasmid construction process.

4. The ligation product was introduced into chemically competent *E. coli* DH5α cells via heat.

5. Transformed colonies on agar plates containing erythromycin were selected. Once recombinant colonies were screened via colony PCR using probe primers, the plasmids from the transformed cells were isolated, and their plasmid DNA was stored at -20°C until needed.

● Note: The recombinant region was confirmed through Sanger sequencing using probe primers to ensure accuracy.

### C. Deletion of the responsible gene in *L. plantarum*

#### C-1. Preparation of competent cells of *L. plantarum*

1. A single colony of *L. plantarum* was inoculated into 3 ml of MRS medium.

2. The overnight culture was diluted 25-fold in MRS supplemented with glycine (1%) and incubated until it reached the exponential phase (OD_600nm_ ~0.6; approximately 3–4 h).

3. Bacterial growth was arrested by incubating on ice for 30 min.

4. Cells were collected via centrifugation at 6,000 × g for 5 min, and the resulting supernatant was discarded.

● Note: Throughout the subsequent steps, cells were maintained at 4°C on ice or in a refrigerated centrifuge.

5. The cell pellet was resuspended in 50 ml of cold 10 mM MgCl_2_ buffer.

6. Centrifugation was performed at 6,000 × g for 5 min, and the supernatant was discarded.

7. The cell pellet was gently resuspended in 25 ml of cold washing buffer (900 mM sucrose and 3.5 mM MgCl_2_ in deionized water).

8. The washing step was repeated twice, using 10 ml of washing buffer for the first wash and 5 ml for the second.

9. The final cell pellet was concentrated in 1 ml of cold washing buffer.

10. The concentrated cells were divided into portions of 80 μl.

● Note: Aliquots were used for electroporation within 1 h on ice or stored at -80°C with 10% glycerol in washing buffer.

#### C-2. Introduction of recombinant plasmid to *L. plantarum*

1. Competent cells (80 μl) were thawed on ice, mixed gently with 1 μg of pGID023-LR plasmid DNA (up to 5 μl), and transferred to a 2 mm electroporation cuvette.

2. The mixture was incubated on ice for 10 min. The electrodes were dried with a paper towel, and electroporation was performed at 1.8 kV.

● Note: A successful electroporation is typically indicated by a time constant ranging from 3.5 to 4.5 ms.

3. The cells were immediately resuspended in 1 ml of pre-warmed recovery broth (MRS media with 0.5 M sucrose and 0.1 M MgCl_2_).

4. The suspension was transferred into a 15 ml test tube and incubated at 37°C for 3 h.

5. 800 μl of the resulting culture was concentrated to 200 μl and spread onto MRS agar plates supplemented with 5 μg/ml erythromycin.

● Note: Transformed colonies typically appear after 1–2 days.

#### C-3. Induction of double homologous recombination

Figure 2 shows a schematic of the minicell purification procedure.

1. The transformed colonies containing the pGID023-LR cointegrate were inoculated into MRS broth supplemented with 5 µg/ml erythromycin and incubated overnight at 37°C.

2. The overnight grown sample (100 μl) was inoculated into MRS broth (10 ml) supplemented with erythromycin (5 µg/ml) and cultured at 37°C overnight.

3. Repeat step 2 seven more times.

● Note: This process is necessary to ensure the plasmid remains integrated into the chromosome; thus, conducting several serial passages of the culture in an erythromycin-containing medium is advisable.

4. The overnight-grown culture (1 ml) was washed twice with fresh MRS broth (1 ml) to remove residual erythromycin. The washed sample (100 µl) was transferred into MRS broth (10 ml) without erythromycin and incubated overnight at 37°C. This process was repeated for ten consecutive passages, each time using MRS broth (10 ml) without erythromycin to facilitate recombination.

● Note: This process promotes intrachromosomal recombination at either the L-arm or R-arm region during cell growth.

5. A stock of the first passage recombinant cultures was prepared and stored at -80°C.

● Note: The first passage recombinant cultures were mixed with glycerol to a final concentration of 20% (cultured cells [500 μl] were combined with 500 μl of 40% glycerol solution).

6. First passage recombinant cultures 1:10^6^ were diluted in MRS broth, and 150 μl of the dilution was spread on MRS agar plate without erythromycin. The plate was incubated at 37°C for 2 days.

7. Colonies that grew in MRS medium but not in MRS medium supplemented with erythromycin were identified.

● Note: At least 50 colonies should be screened because the mutant frequency may vary depending on the target gene and its specific sequence.

● Note: Erythromycin-sensitive clones may arise owing to the second recombination event and can contain either the wild-type or mutant allele.

**Fig. 3.**
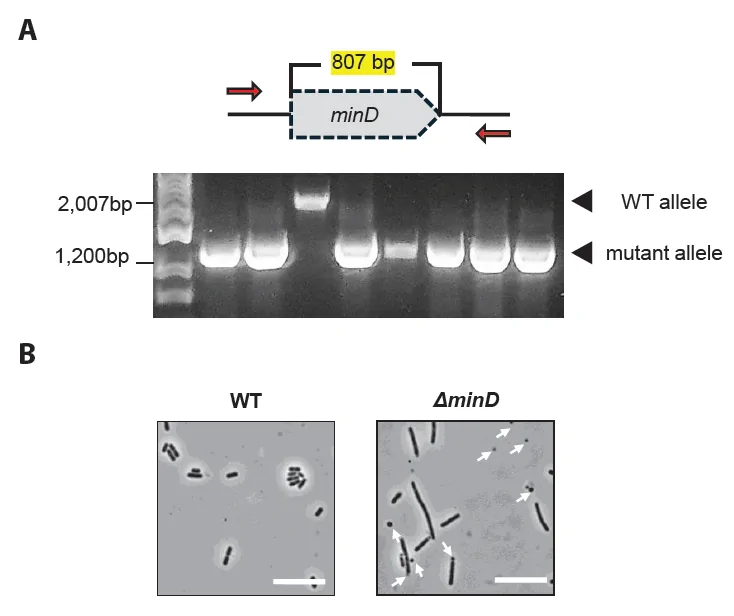
Identification of the deleted *minD* gene in *L. plantarum*. (A) Genetically modified antibiotic-sensitive colonies were validated using PCR to distinguish WT from mutant alleles. (B) Microscopy analysis of the WT and Δ*minD* strains. The cells were cultured overnight in an MRS medium, and the phenotypes of the prepared samples were observed. In the WT strain, uniformly sized rod-shaped cells were observed, whereas the Δ*minD* strain exhibited both elongated cells and small spherical minicells. Minicells are marked with white arrows. Scale bars: 5 μm.

8. The genotypes were validated by performing PCR using the appropriate primer pairs (Fig. 3A).

### D. Isolation and characterization of minicells

**Fig. 4.**
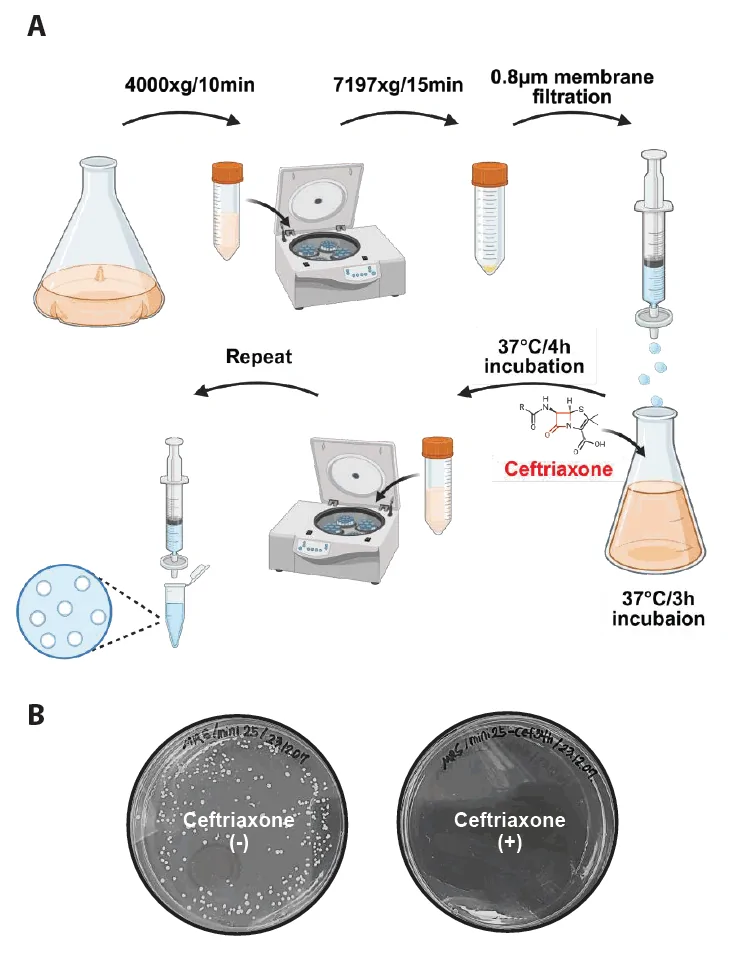
Minicell isolation procedure. (A) The minicell-producing strain was grown in MRS medium until the OD_600_ > 1.0. Larger parent cells were removed by centrifuging at 4,000 × g and 7,197 × g for 10 min and 15 min, respectively, to pellet the minicells. The minicells were resuspended in PBS and filtered (0.8 μm filter) to remove any remaining parent cells. The filtered sample was incubated in fresh MRS medium at 37°C for 3 h, followed by the addition of ceftriaxone (100 μg/ml) and further incubation for 4 h. Centrifugation and filtration was repeated to remove debris and dead cells. The final minicell pellet was washed with PBS and stored at 4°C. (B) Treatment with ceftriaxone enabled the production of highly purified minicells, resulting in minimal presence of parent cells in the isolated sample.

Figure 4A shows a schematic of the minicell purification procedure.

1. A single colony of minicell-producing cells, *L. plantarum* WJL Δ*minD*, was inoculated into MRS broth and incubated at 37°C overnight.

2. A 1000-fold diluted sample was regrown in fresh MRS medium (50 ml) overnight at 37°C.

3. The initial centrifugation of the overnight-grown culture at 4,000 × g was performed for 10 min at room temperature to pellet the parental cells.

4. The supernatant was carefully transferred into new conical tubes (50 ml), and a second centrifugation was performed at 7,197 × g for 15 min to pellet the minicells.

5. The bacterial pellet was resuspended in PBS (500 μl) and filtered through a 0.8 μm filter to remove residual parent cells.

● Note: Culture volume for isolation of minicells can vary depending on the purpose. If the initial culture volume exceeds 1 L, the PBS washing volume should be increased to ensure effective filtration.

● Note: Apply gentle pressure to the syringe to reduce the risk of parental cell contamination. Avoid applying excessive force and stop the process immediately before bubble formation begins.

6. The filtered pellet was resuspended in fresh MRS medium (10 ml) and incubated at 37°C for 3 h to allow recovery.

7. Ceftriaxone was added to the culture to achieve a final concentration of 100 µg/ml. The culture was incubated with ceftriaxone at 37°C for an additional 4 h.

8. Repeat steps 3–5

9. The filtered pellet was transferred to an Eppendorf tube.

10. Centrifugation was performed at 11,000 × g for 5 min using a microcentrifuge.

11. The final pellet was washed in PBS (1 ml).

12. The isolated minicells were stored at 4°C until further use.

● Note: To determine the purity of isolated minicells, microscopy analysis and chromosome staining can be conducted.

## Expected results

The protocol described above enabled minicell production in *L. plantarum* by deleting a putative *minD* gene, which regulates symmetric cellular division (Fig. 3B). This deletion likely regulates the positioning of the FtsZ ring, thereby determining the cell division site, as observed in *B. subtilis* and *E. coli*, where cell division mechanisms are well-defined. Additionally, the protocol includes an optimized method for isolating high-purity *L. plantarum*-derived minicells, highlighting their potential for application in drug delivery systems, where nucleated living bacteria may cause undesirable side effects. This approach demonstrates the feasibility of generating and utilizing minicells in diverse bacterial hosts beyond well-characterized model organisms by disrupting normal cell division through *minD* deletion and efficiently removing parent cells.

Antibiotic treatment enabled the removal of residual parent cells during minicell purification. For instance, ceftriaxone selectively kills actively dividing parent cells during minicell purification by inhibiting cell wall synthesis while leaving anucleate minicells unaffected because of their inability to grow and divide. This ensures a purified minicell preparation, as confirmed by minimal parent cell contamination following drug treatment (Fig. 4B).

**Fig. 5.**
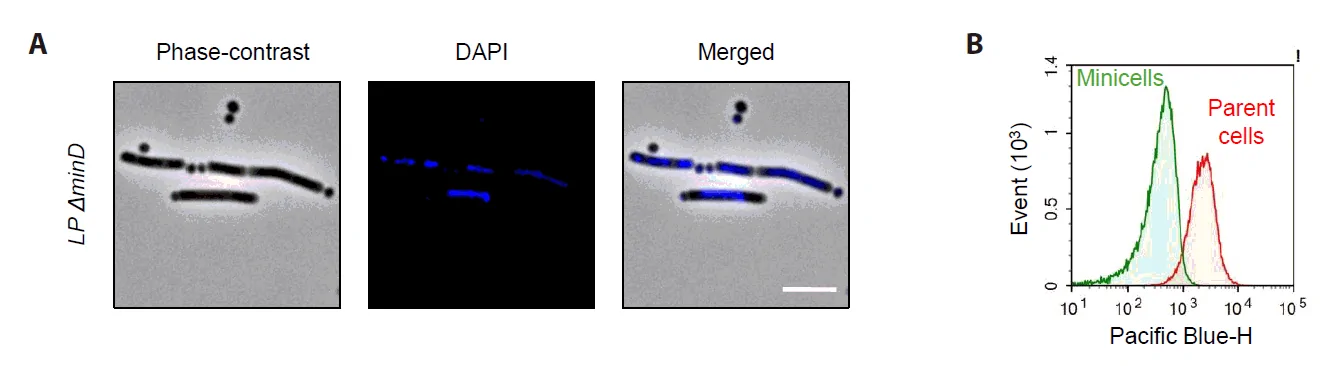
Characterization of *L. plantarum*-derived anucleate minicells. (A) The minicell-producing strain was stained with DAPI to visualize chromosomal DNA. Strong DAPI signals were observed in elongated parent cells, with no detectable signals in the minicells. Scale bars: 2 μm. (B) Flow cytometry analysis of DAPI signals revealed that isolated minicells exhibited significantly lower fluorescence intensities than that in parent cells.

Microscopy and chromosome staining using DAPI were used to characterize anucleate minicells, which showed strong DAPI fluorescence signals in parental cells but no detectable fluorescence signal in small spherical minicells (Fig. 5A). Flow cytometry analysis confirmed distinct fluorescence intensity differences between parental cells and minicells (Fig. 5B). These findings indicate that *Lactobacillus plantarum*-derived minicells lack chromosomal DNA and cannot replicate.

Minicells derived from *Lactobacillus* species are non-pathogenic and safe for therapeutic and probiotic applications. Through engineering, they can display specific surface ligands to enable targeted delivery to cancer cells, infected tissues, or other specific sites while minimizing off-target effects. These features make them valuable for therapeutics, diagnostics, and industrial biotechnology.

## Figures and Tables

**Table 1.** Bacterial strains and plasmids used for this study

Strain or plasmid	Characteristic(s)
Strains	
*E. coli*	
DH5α	Transformation host, erythromycin resistance negative
*L. plantarum *	
WJL	Transformation host, erythromycin resistance negative
Δ*minD*	*L. plantarum *WJL strain with *minD* gene disrupted by double homologous recombination
Plasmids	
pGID023	Shuttle vector for *E. coli *and *L. plantarum*; derivatives of pJDC9 containing the pE194 replication functions; used as an unstable integration vector; Em^r^
pGID023-LR	pGID023 containing the 1,200-bp LR fragment amplified by PCR with the primer L-arm-fw and primer R-arm-rv; Em^r^

**Table 2.** The similarity of the MinD protein identified among various bacterial strains

Strains	*B. subtilis* 168	*E. coli* K-12	*L. pentosus* DSM 20314	*P. putida* NBRC14164	*S. bongori* N268-08	*S. plymuthica* AS9
*L. plantarum* WJL	84.19%	64.46%	99.25%	68.21%	64.69%	65.96%

**Table 3.** Primers used for this study

primer	5’→3’ sequence	Site created
L-arm-fw	cgcaagcttttcgatgatattatgatcgac	HindⅢ
L-arm-rv	aatcaaccgtcaagcctttttcaaacacgtcctccatttc	
R-arm-fw	aaaaggcttgacggttgattaattttcgat	
R-arm-rv	cgcggatccttaatcccagaccaacaacta	BamHⅠ
